# Antimicrobial Usage and Antimicrobial Resistance in Animal Production in Southeast Asia: A Review

**DOI:** 10.3390/antibiotics5040037

**Published:** 2016-11-02

**Authors:** Nguyen T. Nhung, Nguyen V. Cuong, Guy Thwaites, Juan Carrique-Mas

**Affiliations:** 1 Hospital for Tropical Diseases, Wellcome Trust Major Overseas Programme, Oxford University Clinical Research Unit, Ho Chi Minh City, Vietnam; cuongnv@oucru.org (N.V.C.); gthwaites@oucru.org (G.T.); jcarrique-mas@oucru.org (J.C.-M.); 2 Centre for Tropical Medicine, Nuffield Department of Clinical Medicine, Oxford University, Old Road Campus, Roosevelt Drive, Oxford OX3 7BN, UK

**Keywords:** antimicrobial resistance, antimicrobial consumption, antimicrobial residues, animal production, farms, chickens, pigs, *Salmonella*, *E. coli*

## Abstract

Southeast Asia is an area of great economic dynamism. In recent years, it has experienced a rapid rise in the levels of animal product production and consumption. The region is considered to be a hotspot for infectious diseases and antimicrobial resistance (AMR). We reviewed English-language peer-reviewed publications related to antimicrobial usage (AMU) and AMR in animal production, as well as antimicrobial residues in meat and fish from 2000 to 2016, in the region. There is a paucity of data from most countries and for most bacterial pathogens. Most of the published work relates to non-typhoidal *Salmonella* (NTS), *Escherichia coli* (*E. coli*), and *Campylobacter* spp. (mainly from Vietnam and Thailand), *Enterococcus* spp. (Malaysia), and methicillin-resistant *Staphylococcus aureus* (MRSA) (Thailand). However, most studies used the disk diffusion method for antimicrobial susceptibility testing; breakpoints were interpreted using Clinical Standard Laboratory Institute (CSLI) guidelines. Statistical models integrating data from publications on AMR in NTS and *E. coli* studies show a higher overall prevalence of AMR in pig isolates, and an increase in levels of AMR over the years. AMU studies (mostly from Vietnam) indicate very high usage levels of most types of antimicrobials, including beta-lactams, aminoglycosides, macrolides, and quinolones. This review summarizes information about genetic determinants of resistance, most of which are transferrable (mostly plasmids and integrons). The data in this review provide a benchmark to help focus research and policies on AMU and AMR in the region.

## 1. Introduction

Antimicrobial resistance (AMR) is an emerging problem worldwide, and antimicrobial usage (AMU) in animal production is thought to be a contributing factor [1]. Although the magnitude of this has been the subject of much debate, the recent emergence of plasmid-mediated resistance against “last resort” antimicrobials such as colistin from animal production [2] has strengthened the case that AMU in food animal production is a significant contributor to the global burden of AMR in humans [3]. AMU in animal production is likely to contribute to the selection, spread, and maintenance of AMR bacteria on farms. Resistant bacteria, AMR determinants, or the antimicrobials themselves may be disseminated to the environment through farm waste, and may reach humans as a result of direct contact with animals, the consumption of contaminated foods of animal origin, water, and vegetables [4].

Antimicrobials used in animal production and in human medicine are very similar [5], and therefore resistance against antimicrobials of importance for human medicine is of the utmost concern [6]. The high quantities of antimicrobials currently used in animal production are expected to further increase due to increased demand, particularly in emerging economies [7].

Southeast Asia (SEA) is a bloc of rapidly developing and linked economies [8,9]. The area is considered to be a hotspot of AMR [10,11,12]. Some countries in the region have considerably developed their aquaculture (Vietnam, Thailand, Indonesia) and poultry (Thailand) production sectors over recent decades, partly for the export market [13,14]. This represents a further risk of the dissemination of AMR organisms and genes to consumers worldwide.

We reviewed peer-reviewed publications covering antimicrobial usage in animal production, antimicrobial residues, and phenotypic and genotypic AMR traits among bacteria from farms, animals, and food in SEA. Publications investigating AMR against coccidiostats and other antiparasitic, antiviral, or antifungal drugs were not included. The aims were: (1) to describe organisms investigated, sampling/testing methods, host species, as well as country location for phenotypic/genotypic studies on AMR; and (2) to elucidate prevalence as well as country, host species, and secular trends. Studies on zoonotic bacterial pathogens in human communities or clinical settings with no reference to farm/animal/food samples were excluded, as were studies investigating AMR and antimicrobial residues in non-animal samples (i.e., vegetables). By reviewing this information, we aimed to highlight gaps in research that should constitute a basis for the development of policies to control or limit the impact of AMR in the region.

## 2. Results

### 2.1. AMU in Farms

Six publications [15,16,17,18,19,20] reported AMU in terrestrial animal production (pigs and chickens), and two in aquaculture production [21,22]. Except for one publication that investigated antimicrobial use to raise chickens in integrated fish–chicken farms [21] in Thailand, and another one that investigated fish and shrimp farms in both Thailand and Vietnam [22], the remaining publications investigated AMU exclusively in Vietnamese farms. All studies report types and classes of antimicrobials used on farms over a specified period of time. In three publications (two of which relate to the same study farms), quantitative data are also presented [16,18,23]. In two of these studies [16,18] consumption is presented in terms of grams of antimicrobial compound to produce one kilogram of chicken or pig produced, whereas in the other [23], consumption for chickens was expressed as “treatment incidence” [24]. These publications report AMU in 10 different studies (Supplementary Materials Table S1).

The most common antimicrobials reported were amoxicillin (10/10 studies); followed by enrofloxacin, norfloxacin, and doxycycline (9/10 studies); ampicillin and colistin (8/10 studies); neomycin, gentamicin, tylosin, trimethoprim, and florfenicol (7/10 studies); and erythromycin, sulfamethoxazole, and chlortetracycline (6/10 studies).

Three publications from the Mekong Delta of Vietnam have investigated the quantities of antimicrobials administered by the farmer to raise pigs and chickens. Two publications included results from a systematic survey of 208 small (<2000) chicken farms [16], and one reported data from 12 semi-intensive (>500 chickens, >50 pigs) farms [18].

Combined data from these three publications indicate that excluding feed, farmers administered 46 mg per kg of live pig and 52–276 mg of antimicrobial compound per kilogram of live chicken. Interestingly, in smaller chicken farms, the quantities used per chicken produced were higher than in larger farms, even though frequency of usage appears to be lower [16]. Penicillins, lincosamides, quinolones, and combinations of sulphonamides with trimethoprim were quantitatively the most used compounds in Mekong Delta chicken farms, and in 84% of cases, antimicrobials were administered for prophylactic purposes. Pham et al. (2015) reported that 72.3% of aquaculture farms in Vietnam used ~3.3 g of antimicrobial per kilogram of fish/shrimp product; most administered antimicrobials were mixed with the feed [17].

A study reviewed manufacturers′ information on 1462 commercial feed formulations available in Vietnam. The percent of feeds containing antimicrobials ranged from 55.4% (pig), followed by 42.2% (chicken), 18.9% (quail), and 9.2% (duck/Muscovy duck) feeds. Pig feeds had higher contents of antimicrobial than chicken feeds (62.3 mg/kg vs. 25.7 mg/kg, respectively). Overall, it was estimated that 286.6 mg and 77.4 mg of in-feed antimicrobials were used to raise 1 kg of live pig and chicken, respectively. Bacitracin (in 15.5% of formulations), chlortetracycline (11.4%), and enramycin (10.8%) were the most common antimicrobials present in chicken formulations, whereas bacitracin (24.8%), chlortetracycline (23.9%), and florfenicol (17.4%) were the most common in pig feed formulations [19].

In a study in northern Vietnam, pigs were given medicated feed with tetracycline and enrofloxacin on months two and four of the growth phase, and showed concomitant increases in the prevalence of AMR against nalidixic acid and enrofloxacin among both *Escherichia coli* (*E. coli*) and *Enterococcus* spp.. However, there were no appreciable changes in the prevalence of AMR against tetracycline. The study also identified changes in AMR against nalidixic acid and enrofloxacin in isolates from discharge ponds in the same farms [15].

### 2.2. Antimicrobial Residues in Meat/Fish

A study from Vietnam investigated 395 chicken, pork, and beef samples for residues of 21 antimicrobials (six classes) using a liquid chromatography-tandem mass spectrometry (LC-MS/MS) screening method. The percentage of positive samples with detectable residues of at least one antimicrobial were 17.3%, 8.8%, and 7.4% for chicken, pork, and beef samples, respectively. Sulphonamides, fluoroquinolones, and tilmicosin were detected. Sulfaclozine and fluoroquinolones were mainly detected in chicken samples, whereas sulfamethazine was mainly detected in pork samples [25]. Another study from Vietnam investigating fluoroquinolone and tetracycline residues in 104 fish/shrimp retail samples concluded that overall 27% of retail samples tested positive for either of the two, using a range of techniques [17]. A further study in Vietnam investigated 290 pork samples for tetracycline residues. Residues were detected in about 5.5% of all analysed samples, with samples from different geographic areas sampled showing considerable differences in prevalence [26].

### 2.3. Phenotypic AMR among E. coli and Non-Typhoidal Salmonella (NTS)

A total of 39 publications investigated phenotypic AMR traits among NTS (30) and “commensal” *E. coli* (12) isolates from animal sources. In three publications, AMR was investigated in both *E. coli* and NTS isolates. The majority of publications included data from Thailand (20) [27,28,29,30,31,32,33,34,35,36,37,38,39,40,41,42,43,44] and Vietnam (15) [18,23,45,46,47,48,49,50,51,52,53,54,55]. One publication was found with data from each of Cambodia, Indonesia, Lao PDR and Malaysia [56,57,58,59]. One study investigated imported aquaculture products from several SEA countries [60]. A total of 14/39 publications investigated AMR from more than one type of animal, 9 of which included chickens and pigs. The species investigated were—in decreasing order—pigs/pork (27 publications), chickens (18), ducks (4), aquaculture (5), cattle/beef (3), unspecified poultry (3), and wild mammals (1).

**Figure 1:**
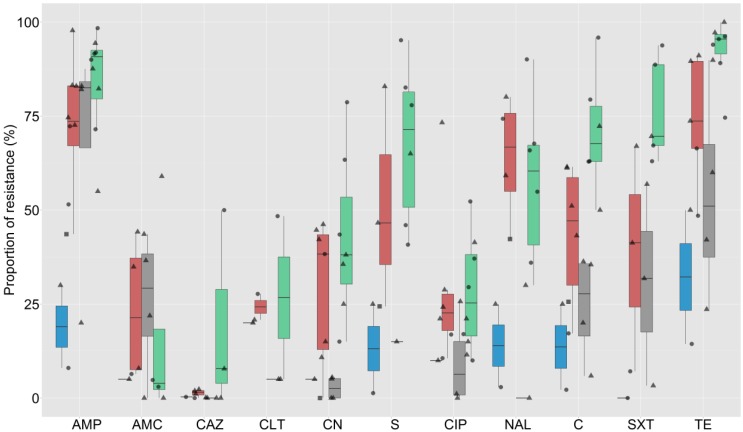
Summary results of estimates of prevalence of antimicrobial resistance (AMR) in *Escherichia coli* isolates of animal origin (N = 23). Blue = aquaculture, Red = chicken, Green = pig, Grey = other species. Circle = Thailand, triangle = Vietnam, square = Indonesia. Key: AMP = ampicillin, AMC = augmentin, CAZ = ceftazidime, CLT = cephalothin, CN = gentamicin, S = streptomycin, CIP = ciprofloxacin, NAL = nalidixic acid, C = chloramphenicol, SXT = co-trimoxazole, TE = tetracycline. Boxplots indicate median and 75% interquartile range.

**Figure 2:**
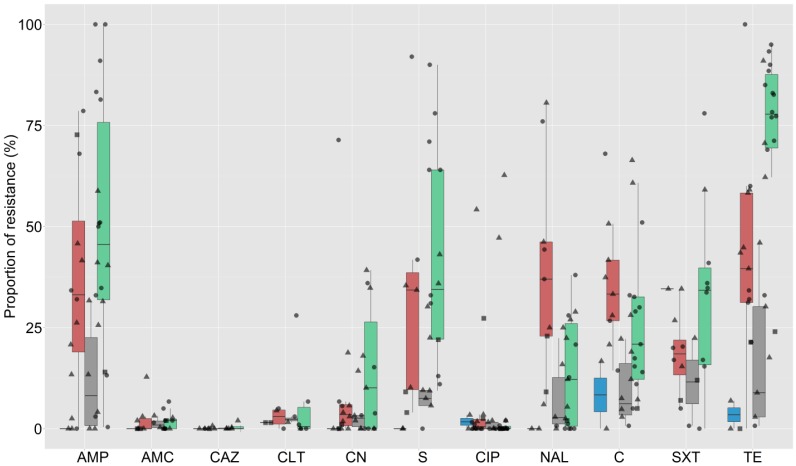
Summary results of estimates of prevalence of AMR in non-typhoidal *Salmonella* (NTS) isolates of animal origin (N = 41). Blue = aquaculture, Red = chicken, Green = pig, Grey = other species. Circle = Thailand, triangle = Vietnam, square = other countries (Cambodia, Lao PDR, and Malaysia). Key: AMP = ampicillin, AMC = augmentin, CAZ = ceftazidime, CLT = cephalothin, CN = gentamicin, S = streptomycin, CIP = ciprofloxacin, NAL = nalidixic acid, C = chloramphenicol, SXT = co-trimoxazole, TE = tetracycline. Boxplots indicate median and 75% interquartile range.

The testing methods employed included disk diffusion (26 publications), broth microdilution (7), and agar dilution (6). A total of 3598 *E. coli* and 4726 NTS strains were investigated in these studies. The criteria for breakpoint interpretation of all studies were Clinical Laboratory Standard Institute (CLSI) guidelines, except for two studies where, depending on the antimicrobial, the interpretation criteria included both CLSI and European Committee on Antimicrobial Susceptibility Testing (EUCAST) guidelines. In one study, the interpretation criteria were not provided by the authors. Data from the publications were extracted to generate 73 prevalence estimates of AMR against different combinations of antimicrobials among *E. coli* and NTS isolates from animals. In total, 32 different antimicrobials were tested across all studies, with a median of 10 (interquartile range (IQR) 8–10) antimicrobials tested. The most common antimicrobials investigated were ampicillin (69 estimates), tetracycline (64), chloramphenicol (61), ciprofloxacin (61), gentamicin (59), streptomycin (49), nalidixic acid (49), amoxicillin/clavulanic acid (37), and co-trimoxazole (33). The overall estimates for AMR in chickens, pigs, aquaculture, and other host species among studies for the 11 most common antimicrobials tested are shown in Figure 1 and Figure 2 for *E. coli* and NTS isolates, respectively. Data from two publications including AMR phenotypic results for NTS isolates from pigs and poultry combined [30,42] were not plotted.

The median (and IQR) prevalence of AMR against all tested antimicrobials by country and species is shown in Supplementary Table S2 (NTS) and Table S3 (*E. coli*). Across studies, the median prevalence of resistance among *E. coli* isolates was >70% for ampicillin and tetracycline; >50% to 70% for sulphamethoxazole, amoxicillin, co-trimoxazole, and nalidixic acid; >20% to 50% for trimethoprim, chloramphenicol, streptomycin, enrofloxacin, gentamicin, ciprofloxacin, colistin, and cephalothin; >10% to 20% for norfloxacin and kanamycin; >1% to 10% for augmentine, ceftiofur, and cefotaxime; and 0.1% to 1% for ceftriaxone and ceftazidime.

The median prevalence of resistance among NTS isolates was >20% to 50% for tetracycline, spectinomycin, sulphamethoxazole, trimethoprim, ampicillin, streptomycin, amoxicillin, and co-trimoxazole; >10% to 20% for chloramphenicol, florfenicol, and nalidixic acid; >1% to 10% for neomycin, gentamicin, augmentin, kanamycin, cephalothin, and amikacin; and 0.1% to 1% for colistin. Resistance against ceftazidime, ceftiofur, ceftriaxone, norfloxacin, ciprofloxacin, and cefotaxime was <0.1%. In all studies investigating phenotypic resistance against third-generation cephalosporins in NTS strains, the prevalence found was <5%, except for two studies in Thailand, where cefotaxime prevalence in pig isolates was ~12% [38,42].

The raw data alongside the summary prevalence of resistance for the 10 most common NTS serovars for which AMR data are available are presented in Supplementary Materials Table S4. *Salmonella typhimurium* isolates displayed resistance against the highest number of antimicrobials (over 30% resistance for ampicillin, streptomycin, gentamicin, tetracycline, and nalidixic acid), followed by *Salmonella rissen* (over 30% resistant strains against ampicillin, sulphamethoxazole, and tetracycline).

Results from multivariable models investigating resistance against the five most commonly investigated antimicrobials are presented in Table 1. Compared with chicken isolates, isolates from pigs had significantly higher probability of being resistant against all five antimicrobials investigated (OR ranging from 1.47 to 6.67), and isolates from other terrestrial animals (cattle, buffaloes, and ducks) and aquaculture production had a lower probability of resistance against most antimicrobials tested (ampicillin and gentamicin resistance in NTS isolates from aquaculture could not be modelled). Isolates from Thailand and Vietnam had overall higher probability of resistance compared with isolates from the other countries combined (however, comparisons for resistance against gentamicin, tetracycline, and ciprofloxacin with *E. coli* isolates from other countries could not be drawn). Isolates displayed a considerable increase in resistance against ampicillin, chloramphenicol, and ciprofloxacin over the 2008–2016 period compared with studies carried out earlier than 2008, both for *E. coli* and NTS, but no difference or a reduction of resistance was seen in the case of gentamicin (Table 1).

Five publications (three from Vietnam, two from Thailand) compared phenotypic AMR profiles between human and animal isolates. Three publications involved the investigation of NTS isolates from diarrhoea patients in unrelated hospitals [31,45,48]. Two publications investigated AMR in farmers (one investigating *E. coli* and one NTS) [29,55]. A study of NTS from chicken flocks (164 isolates) and their asymptomatic farm owners and non-farming control in the same areas (17 isolates) showed higher levels of AMR among NTS isolates from chickens except for ceftazidime (a third generation cephalosporin), an antimicrobial where the prevalence among 17 human isolates was 6% compared to 0% among 164 chicken isolates [55]. Another study compared a subset of study *E. coli* isolates (including Class 1 integron-positive) from pigs and farmers (62 and 7, respectively), and found overall higher levels of AMR among pig isolates, including resistance against cephalothin (a first generation cephalosporin) [29]. The comparisons in the prevalence of AMR among NTS isolates from human patients in Vietnam and Thailand with animal and food isolates show highly variable results [31,45,48].

### 2.4. Phenotypic AMR among Campylobacter spp.

Eighteen publications investigated phenotypic AMR traits among *Campylobacter* spp. isolates from animal sources, including poultry (16 publications) [41,58,61,62,63,64,65,66,67,68,69,70,71,72,73,74], pigs (4) [64,68,70,75], and aquaculture products (1) [76]. Three publications reported phenotypic AMR profiles from both poultry and pig isolates. The majority of publications were from Thailand (10), followed by Vietnam (4), Malaysia (2), Cambodia (1), and the Philippines (1). The methods used for phenotypic antimicrobial susceptibility testing included disk diffusion (8 publications), broth microdilution (6), E-test (3), and agar dilution methods (1). The antimicrobial susceptibility profiles were analysed based on the guidelines from CLSI (10 publications), National Antimicrobial Resistance Monitoring System (NARMS) (2), EUCAST (1), Comité de I’Antibiogramme de la Société Française de Microbiologie (CASFM) (1) and not reported (3). In one study, the interpretation of breakpoints included both CLSI and NARMS guidelines. In total, 2355 *Campylobacter* spp. isolates were tested, resulting in 25 estimates of AMR prevalence. In most studies (except two) [71,76], the strains were species identified (either by polymerase chain reaction (PCR) or by phenotypic methods).

Among 20 different antimicrobials tested, the most common substances investigated were ciprofloxacin (23 estimates), erythromycin (22), gentamicin (17), tetracycline (16), nalidixic acid (14), chloramphenicol (13), and ampicillin (10). The levels of AMR among *Campylobacter* spp. from SEA publications are presented in Table 2.

Overall levels of AMR were >70% for nalidixic acid, tetracycline, and ciprofloxacin; >50% to 70% for streptomycin; >20% to 50% for erythromycin, ampicillin, and co-trimoxazole; >10% to 20% for gentamicin; and >1% to 10% for azithromycin and chloramphenicol. Generally, isolates from Vietnam displayed higher levels of resistance compared to Thailand, except for ciprofloxacin (79.6% resistance among Thai isolates vs. 44.5% among Vietnamese isolates) and tetracycline (77.2% resistance among Thai isolates, 73.0% Vietnamese isolates).

*Campylobacter* spp. isolates from pigs displayed generally higher levels of resistance compared with those from poultry for ampicillin (51.2% vs. 37.1%), tetracycline (84.2% vs.71.0%), nalidixic acid (89.6% vs. 83.3%), ciprofloxacin (84.5% vs. 66.6%), and especially for erythromycin (66.0% vs. 23.7%). A study of AMR in *Campylobacter* spp. in commercial broilers showed that levels of AMR were generally higher among older birds [65]. Study on mollusks showed that *Campylobacter* spp. isolates were highly resistant to erythromycin (77.1%), nalidixic acid (32.7%), and ciprofloxacin (23.4%) [76].

### 2.5. Phenotypic AMR in Bacteria from Aquaculture

A total of eight publications investigated phenotypic AMR traits in bacteria isolated from aquaculture products (other than *E. coli*, NTS, and *Campylobacter* spp.) [21,77,78,79,80,81,82,83]. These publications included research related to fish (6) and shrimp farms (2). All publications came from Thailand and Vietnam, even though four investigated aquaculture samples originating from several SEA countries (Thailand, Vietnam, Malaysia, and Indonesia) [78,79,80,82]. One study investigated AMR in isolates from imported aquaculture products from Thailand. A total of 817 isolates belonging to seven bacterial species were investigated, including fish pathogens (*Edwardsiella ictaluri*, *Streptococcus agalactiae*, *Streptococcus dysgalactiae*, and *Klebsiella* spp.), and “commensal” or environmental bacteria (*Enterococcus* spp., *Aeromonas* spp., and *Pseudomonas* spp.) (Table 3).

The testing methods included disk diffusion (seven publications) and agar dilution test (1). In all cases, CSLI guidelines for breakpoint interpretation were followed. The most common antimicrobials investigated were chloramphenicol (11 estimates), oxytetracyline (8), streptomycin (7), ampicillin (6), erythromycin (7), ciprofloxacin (6), and co-trimoxazole (5).

Results from these studies are highly variable, given the diversity of organisms and antimicrobials investigated (Table 3). A Thai study showed higher levels of resistance in *Enterococcus* spp. isolates in the intestinal contents of fish from integrated fish-chicken farms, compared with a control fish farm not using chicken manure to fertilise the pond. However, no significant difference in levels of AMR was found in *Aeromonas* spp. isolates in both types of farm [21]. A Vietnamese study investigated AMR among the common pathogen *Edwardsiella ictaluri* (causative of bacillary necrosis of *Pangasius* fish) during the period 2002–2005. Acquired resistance to each of streptomycin, oxytetracycline, and trimethoprim was detected in >70% of the isolates. However, resistance against beta-lactams, aminoglycosides, and quinolones was <5% [77].

### 2.6. AMR in Bacteria from Terrestrial Animals Other Than E. coli, NTS, and Campylobacter spp.

A total of 19 publications investigated phenotypic AMR in organisms other than *E. coli*, NTS, and *Campylobacter* spp. in terrestrial animals (Table S5). A total of nine studies investigated *Enterococcus* spp., including data from Malaysia (4) [84,85,86,87], Thailand (3) [40,57,88], Vietnam, and Indonesia (1 each) [57]. These publications investigated chicken or chicken meat samples [57,84,85,87], two investigated pigs [40,88], and one investigated beef samples [86]. Two publications included isolates randomly selected from meat/farm samples [40,57], whereas five studied vancomycin-resistant *Enterococcus* spp. (VRE); bacteria were directly isolated by using selective plates [84,85,86,87,88]. Among the five VRE studies, *Enterococcus faecalis* was the predominant *Enterococcus* spp. in chickens [84,85,87], whilst *Enterococcus gallinarum* was most prevalent in pigs [88]. In general, levels of resistance among VRE strains were greater than *Enterococcus* spp. isolates randomly selected from the same chickens, especially for aminoglycosides, tetracylines, quinolones, and macrolides [57,87]. In one study, VRE carriers were found in ~25% of pigs of all age groups, and a large proportion of VRE were also resistant to tetracycline (86.5%) [88]. In another study, all 12 *β*-hemolytic vancomycin-resistant *E. faecalis* isolates recovered from beef samples were resistant to nine antimicrobials tested [86].

In five publications from Thailand, methicillin resistant *Staphylococcus aureus* (MRSA) was investigated in pigs and pork meat [89,90,91,92,93]. In one study, a total of 104 pig farms were investigated for MRSA, alongside farmers and the farms′ environment. The herd-level prevalence of MRSA was ~9.6%, and the individual pig and farmer prevalence was 0.7% and 2.5%, respectively [93]. All (100%) MRSA isolates were resistant to clindamycin, oxytetracycline, and tetracycline, while 100% were susceptible to vancomycin [92,93]. The Staphylococcal Cassette Chromosome *mec* (SCC*mec*) clonal type IX appears to be quite disseminated in the Thai pig industry, representing a reservoir for human infection [91,92]. A study in Vietnam investigated AMR in 45 *Streptococcus suis* serotype 2 isolates from pig tonsils. All isolates were fully susceptible to penicillin, vancomycin, and ciprofloxacin, but 51% were resistant to erythromycin, and all were resistant to tetracycline [94].

Three publications have investigated AMR in poultry pathogens, including *Mycoplasma gallisepticum*, *Haemophilus paragallinarum*, and *Avibacterium paragallinarum* [95,96,97], two in clinical and animal *Burkholderia pseudomallei* isolates [98,99], and one in *Clostridium perfringens* bacteria from diarrhoeic pigs [100].

### 2.7. Genotypic Studies

Thirteen publications reported plasmid-mediated *β*-lactamase-encoding genes among *E. coli* and NTS isolates from pigs (11 publications) [32,36,39,46,48,101,102,103,104,105,106], poultry (8) [32,46,48,101,102,103,104,105], cattle (4) [46,48,49,104], and aquaculture (4) [46,101,104,105]. A total of nine different genes have been described in isolates from Thailand, Vietnam, Malaysia, Lao PDR, and Indonesia (*bla*_TEM_, *bla*_PSE-1_, *bla*_CTX-M_, *bla*_SHV_, *bla*_OXA_, *tem*A, *tem*B, MOX, and DHA). While TEM, SHV, and CTX-M were frequently detected in *E. coli* isolates, TEM, OXA and PSE-1 were often reported among NTS isolates (Table 4).

CTX-M and TEM were the most common *β*-lactamase-encoding genes identified among ampicillin-resistant and/or ESBL-producing *E. coli* isolates [39,105]. CTX-M-1 and CTX-M-9 were the main CTX subgroups observed in *E. coli* isolates from chicken meat and pork (Vietnam) and healthy swine (Thailand) [39,105]. In addition, CTX-M-55 was also found among colistin-resistant *E. coli* isolates from Vietnam [106]. PCR and sequence analysis revealed that extended spectrum beta-lactamase (ESBL)-positive *E. coli* isolated from healthy pigs in Thailand carried TEM-1, TEM-135, and TEM-176 subgroup genes [39]. To our knowledge, no studies have investigated the presence of ESBLs in NTS from meat or animals.

Quinolone resistance among Enterobacteriaceae conferred by mutations of the *gyr*A and *gyr*B genes was a consistent finding among various studies in Thailand and Vietnam [18,32,79]. Plasmid-mediated resistance genes were also detected among *E. coli* and NTS isolates; however, they had less contribution to high levels of nalidixic acid and ciprofloxacin resistance [18,107]. While mutations in 23S rRNA were confirmed as the most common mechanism for macrolide resistance in *Campylobacter* spp. [70,75], the possession of *erm* genes was predominantly associated with resistance among *Enterococcus* spp. and *S. suis* isolates [108,109].

Since the first description of a plasmid-mediated colistin resistance gene (*mcr-1*) in 2015 in China, several studies from SEA have investigated this gene in Enterobacteriaceae. The occurrence of *mcr*-1 positive isolates of livestock has been documented in Vietnam and Lao PDR [18,106,110]. A study of 180 *E. coli* isolated from pig and chicken farms in Vietnam showed a 18.9%–22.2% prevalence of the *mcr*-1 gene, and a high level of agreement with phenotypic colistin resistance [18]. Both *mcr*-1 sequences from Vietnam and Lao PDR were identical to the gene reported in China [2].

A total of 12 publications reported the presence of class 1 integrons among *E. coli* and NTS isolates in SEA countries. The majority of studies investigating class 1 integrons came from Thailand (9) [29,32,34,35,36,38,39,103,111], followed by Vietnam (2) [48,112] and Malaysia (1) [104]. Aggregated data from all studies indicate that 1095 NTS and 1094 *E. coli* isolates were investigated for the presence of integrons by PCR amplification of the *intI* gene. Generally, class 1 integrons were found at higher prevalence among *E. coli* isolates (median of 72.1%), compared with NTS isolates (median of 27.0%). The gene cassettes found in class 1 integrons carried by *E. coli* and NTS included the *aadA*1, *aad*A2, *aad*A3, *aad*A4, *aad*A5, *aad*A22, *aad*A23, and *aac*A4 (conferring resistance to aminoglycosides), *dfr*A1, *dfr*A5, *dfr*A12, *dfr*A17, *dhfrXII*, and *dhfrA17* (diaminopyrimidines), *bla*_PSE-1_ and *bla*_OXA-30_ (*β*-lactams), *lin*F and *lun*F (lincosamides), *cat*B3 (chloramphenicol), and *sul*1, *sul*2, and *sul*3 (sulphonamide) genes.

## 3. Discussion

Across SEA countries, there is great variability in the published research on AMU/AMR in animal production. Most publications focused on NTS, *E. coli*, and *Campylobacter* spp., and to a lesser extent *Enterococcus* spp. and MRSA. This is likely to reflect existing research capacity as well as public health priorities. NTS is considered one of the most common foodborne bacterial pathogens in the region [113,114]. In addition, the spread of NTS has been linked to international travel and global food trade [115]. The fact that Thailand is a major tourist destination as well as a global exporter of poultry and aquaculture probably explains the larger number of published research on NTS from that country. In contrast, there is a scarcity of published data from large countries such as Indonesia, Myanmar, and the Philippines.

The scarcity of reliable quantitative and qualitative data on AMU in different animal production systems across the region is a major gap in the research. Accurate AMU data are difficult to gather and require labour-intensive surveys in the absence of reliable figures on antimicrobial sales. Qualitative data available (mostly AMU frequency of specific antimicrobials) from specific studies suggests a very high diversity of antimicrobials used both as growth promoters (AGPs), as well as for prophylactic and treatment purposes, although results are difficult to compare across studies. It is of concern that the usage of chloramphenicol has been reported on pig and poultry farms in Vietnam [20,51], even though the use of this antimicrobial has been banned in the country for more than 15 years. Although there are no published data from SEA countries other than Vietnam, the situation is probably similar. There is also potentially an issue with regards to the types and quantities of APGs used. In the EU, a total of 11 non-ionophore AGPs have been used historically, with no more than three to four antimicrobials available at any given time, none of them listed by WHO as “critically important” [116]. In contrast, a recent study of commercial pig and poultry feed products in Vietnam found 14 different AGPs that included colistin, amoxicillin, and neomycin [19].

Because of the considerable differences in production systems, sampling protocols, as well as differences in laboratory testing methods, phenotypic estimates of AMR prevalence in this review need to be interpreted with great care. In most cases, specimens were collected using “convenience sampling”, and studies probably have a large sampling error. By aggregating the data, we hoped to reduce this error. The interpretation of what constitutes a resistant or susceptible strain is a function of the methodology used and the interpretation criteria. The EU monitoring data uses mostly EUCAST epidemiological breakpoints, which do not necessarily correspond to CLSI guidelines. In particular, the definition of the intermediate category is likely to be the source of important discrepancies [117]. In most of the publications, the authors do not make clear whether the prevalence of resistance relates to strictly resistant strains or resistant plus intermediately resistant.

Among NTS and *E. coli* strains, the prevalence of AMR against “older” antimicrobial classes such as ampicillin, tetracycline, and sulphonamide tended to be high or extremely high (i.e., ampicillin and tetracycline in *E. coli*). Resistance against quinolones (ciprofloxacin, enrofloxacin), aminoglycosides (gentamicin), and polymixins (colistin) among *E. coli* isolates is also a concern (>20% to 50% resistance). Overall, NTS levels of resistance were lower (<20%) for aminoglycosides and quinolones, and very low/rare for third generation cephalosporins and colistin. Resistance against third generation cephalosporins was, however, low or very low (<10%). Compared with EU data, NTS and *E. coli* isolates had higher levels of resistance for most types of antimicrobials. The most relevant exception is nalidixic acid, ciprofloxacin (quinolones) (chickens), and third generation cephalosporins (ceftriaxone) (chickens and pigs), where levels of resistance are higher in EU isolates. Results from *Campylobacter* spp. are striking for the higher prevalence of resistance against ciprofloxacin (median 70.4%) and erythromycin (median 46.2%). The observed levels of resistance against ciprofloxacin were not dissimilar to *C. jejuni* and *C. coli* isolates isolated from chicken meat (2013) (53.0% and 76.2%) in the EU, but much higher than for erythromycin (<11%) [118].

Increased resistance to third-generation cephalosporins—including resistance conferred by extended spectrum beta-lactamases (ESBLs) and resistance against fluoroquinolones in Enterobacteriaceae—are of great concern worldwide [12]. Although we found evidence of ESBL in all types of animal production, it is not clear whether the prevalence of ESBL-positive bacteria is particularly high in SEA animal production [39,105], in spite of evidence of high prevalence in human populations [119,120]. The data reporting ESBL-positive *E. coli* from samples using selective plates cannot be compared with individual colony-level prevalence. For example, in a survey where 208 Vietnamese chicken farms were investigated by culturing pooled faecal samples using selective plates containing ceftazidime, the sample-level prevalence was 14.9%, compared with a 0.2% prevalence of ESBL-producing *E. coli* colonies [23]. Data from the same study indicated that the prevalence of ESBL among *E. coli* colonies from farmers (31.1%) and other non-farming human communities was much higher (38.3%–49.5%) than among isolates from chickens (20.0%), due to extensive use of cephalosporins by the human subjects [119].

More recent studies have included colistin in the testing panel for *E. coli* isolates. The data from animals from Thailand and Vietnam suggest that resistance levels against this antimicrobial in *E. coli* are on the order of 15%–25% in poultry and pigs. Studies in the region have also shown the widespread presence of genes encoding for colistin resistance in human populations in Cambodia, Lao PDR, Thailand, and Malaysia [110,121,122]. Recent data from EU monitoring from 2014 indicate low-level resistance in 8% (minimum inhibitory concentration of 4–8 mg/L) *E. coli* isolates. The recent increase in reports of invasive infections caused by *mcr-*1 plasmid-mediated colistin resistance worldwide is of great concern [123]. Since this antimicrobial is currently used as AGP in Vietnam (and possibly in other countries), the restriction of this antimicrobial should be a priority [19]. Data is not available on carbapenem resistance testing among isolates in the region. However, to our knowledge, carbapenems are not licenced nor used in agriculture in the region. Most integrons could be horizontally transferred to other strains by conjugation [34,36,48,103,111], suggesting that class 1 integrons probably also have a large contribution in acquisition and the widespread antimicrobial resistance profiles [34]. The identical transferable elements found in bacterial isolates from animals and humans and food-producing animals are believed to reflect dissemination of resistant bacteria and resistant genes across species [124].

After adjusting for country and time of study, *E. coli* and NTS isolates from pigs displayed higher levels of AMR against five antimicrobials investigated compared with other species. The reported low levels of AMR in *E. coli* and NTS from aquaculture is intriguing. Neither NTS nor *E. coli* are considered to be commensal organisms of fish/shellfish. Therefore, the isolation of these species is likely to reflect contaminated water with excreta, the use of contaminated feed, or post-harvest contamination. In many farms in the region, animal excreta are commonly-used fertilisers of ponds to support the growth of algae that will be a source of feed to fish. One study analysed AMR in flora from probiotics used for shrimp production, and concluded that the overall levels of AMR in isolates from such products are generally low [52]. It is also possible that some isolates recovered from shrimp may have originated from sources other than the shrimps themselves (i.e., wild fauna, humans, other animals). It has been shown that *E. coli* isolates from wildlife have generally lower levels of AMR compared with farmed species [51].

We recommend caution when interpreting results from modelling aggregated data from such a diversity of studies, given the variability of sampling conditions and laboratory techniques.

AMR in NTS is known to be partly related to serovar identity. However, a study in Vietnam found that NTS isolates from pig species had a higher prevalence of multidrug resistance after adjusting for serovar [53]. It is not known for certain whether this is a reflection of differences in levels of AMU between the two species.

Studies from Vietnam do not suggest large differences between the quantities of antimicrobials administered by the farmers in chicken and pig production. However, compared with chickens, pigs in Vietnam are raised using considerably higher amounts of antimicrobials in feed. The longer cycle of pig production, resulting in increased exposure to antimicrobials, and the difficulties associated with cleaning and disinfection and persistence of certain resistant clones in pig farms may also contribute to the observed differences in AMR prevalence [19]. Similar findings can be described for *Campylobacter*, where isolates from pigs also had consistently higher levels of resistance than isolates from poultry. The most remarkable difference relates to erythromycin resistance. It is speculated that this derives after decades of use of macrolides such as tylosin in the pig industry [125], which has been associated with concomitant resistance to erythromycin [126].

Our analyses conclusively indicate that there has been a considerable (and statistically significant) increase in AMR over recent years among Enterobacteriaceae. This is most evident for antimicrobials such as ampicillin, ciprofloxacin, and chloramphenicol.

AMR in VRE strains has been investigated in a number of countries because of the concerns for invasive human infections with a concern in the EU in association with the use of avoparcin (a glycopeptide analogue) as a growth promoter in the 1990s and early 2000s, before it was banned [127]. The use of avoparcin as AGP is not well documented in the SEA region. However, a recent survey of commercial feeds in Vietnam did not identify this antimicrobial in any of the formulations examined [19].

A major gap is the scarcity of data on the prevalence of AMR in animal pathogens. Most of the research carried out on AMR in animal production responds to human health concerns. However, better awareness of the consequences of AMR in disease control and farm productivity would be a useful incentive to restrict the unnecessary use of antimicrobials in animal production.

Measuring AMR in aquaculture is challenging because of the lack of consensus on a universal “indicator” organism. A study indicated that whereas *Enterococcus* spp. appeared to reflect AMU selection pressure on farms, this was not the case for *Aeromonas* spp. [21]. Clearly, aquaculture represents a crucial type of animal production in SEA, with countries such as the Philippines, Thailand, Vietnam, and Myanmar being major fish exporters [128]. Quantitatively, the use of antimicrobials in aquaculture appears to be of much greater magnitude compared with terrestrial animals. The impact of AMU and AMR in animal production goes beyond the sphere of human health and food safety. In addition to AMU in aquaculture, the common practice of discharging manure from terrestrial animals into water systems leaves the aquatic environments in SEA particularly vulnerable to the development of AMR [129]. More research is clearly needed to investigate the impact of AMU/AMR in farming systems on environmental microbiota.

## 4. Materials and Methods

We searched PubMed for publications using the following combinations of terms in either the title or the abstract: (1) “Antibiotic use”, “antimicrobial use”, “antimicrobial usage”, “antibiotic use”, “antimicrobial consumption”, “antibiotic consumption”, “antimicrobial residues”, “antibiotic residues”; (2) “poultry”, “chicken”, “duck”, “pig”, “fish”, “cattle”, “buffalo”, “aquaculture”, “shellfish”, “shrimp”, “wildlife”, and “animal production”; (3) Southeast Asia, Thailand, Vietnam, Lao PDR, Cambodia, Myanmar, Indonesia, the Philippines, Malaysia, East Timor, Brunei, and Singapore. The time period was from 2000 to July 2016. Studies exclusively investigating the prevalence/presence of infection or contamination with bacterial strains were only included if they provided information regarding the prevalence of AMR.

In order to measure the independent contributions of country, host species, and time period on the observed differences, multivariable logistic regression models were built investigating the outcome “probability of resistance” against the most commonly tested antimicrobials for *E. coli* and NTS isolates using prevalence estimates as reported in publications. The prevalence data reported, alongside the number of colonies investigated in each study were used to generate “individual colony” datasets that were readily used for modelling. No further adjustment was carried out, and therefore larger studies carried more weight in the analyses. The explanatory variables fitted were: “country” (Thailand, Vietnam, and “other countries”), “species” (“pigs”, “chicken”, “aquaculture”, and “other”), and time (study conducted before or after 2007). The analyses were weighted to reflect the variable sample sizes of different studies. All statistical analyses were carried out using R statistical software version 3.3.1 (The R Foundation for Statistical Computing, Vienna, Austria).

The thresholds used to summarise levels of AMR were: <0.1%, 0.1% to 1%, >1% to 10%, >10% to 20%, >20% to 50%, >50% to 70%, and >70% based on European Food Safety Authority (EFSA) guidelines [130].

## 5. Conclusions

This review has highlighted the considerable gaps in data on antimicrobial consumption in farming systems in the region, as well as large variation in methodologies and data available for phenotypic AMR testing. Differences in perceptions and concerns among the different parties involved in AMU/AMR in animal production in the region represent a formidable challenge for concerted action to limit its impact. Given the globalization of the antimicrobial markets, it would be desirable to move towards the harmonization of surveillance systems to monitor AMU in animal production, as well as for testing animal products for AMR and antimicrobial residues in foods of animal origin. Of particular urgency is the implementation of policies that restrict the use of antimicrobials of critical importance. The use of AGPs has recently been the subject of much discussion in the region. Recently, Thailand took a major bold step by banning the inclusion of AGP since 2015 [131]. It is hoped that other countries will follow suit, and the momentum is increased for implementing policies that restrict the use of certain antimicrobials in animal production in the region. It is expected that this will be facilitated, given the stated drive towards a single market and equitable economic development within the Southeast Asian region [132].

## Figures and Tables

**Table 1:** Odds ratios (ORs) derived from multivariable logistic regression models investigating risk factors for positivity among *Escherichia coli* and non-typhoidal *Salmonella* (NTS) isolates against five antimicrobials in Southeast Asia (SEA). The reported *p*-values refer to the significance levels of the investigated variable.

Variable	*Escherichia coli*					Non-typhoidal *Salmonella*				
	AMP (3294) ^§^	CN (2667)	C (2799)	TE (3231)	CIP (2857)	AMP (3849)	CN (3144)	C (3339)	TE (3352)	CIP (4026)
Host type (baseline = Chicken)										
Aquaculture	0.03 ***	0.16	0.04 ***	0.12 ***	0.71	NC	NC	0.15 **	0.05 ***	0.73
Pig	2.33 ***	2.35 ***	3.26 ***	6.65 ***	1.45 **	2.86 ***	3.98 ***	1.46 ***	3.89 ***	3.19 ***
Other ^a^	0.93	0.17 ***	0.29 ***	0.17 ***	0.43 ***	0.32 ***	0.54 *	0.31 ***	0.21 ***	0.33 **
Country (baseline = Other ^b^)										
Thailand	1.75 *	1 (baseline)	1.98	1 (baseline)	1 (baseline)	2.52 ***	8.16 **	7.55 ***	4.12 ***	0.02 ***
Vietnam	2.19 **	0.44 ***	2.87 **	5.31 ***	0.91	1.18	5.40 *	11.10 ***	4.10 ***	0.41
Period (baseline = 2007 or earlier)										
2008–2016	2.93 ***	0.68 *	1.39 *	0.82	2.46 ***	3.73 ***	1.12	1.39 ***	1.38 **	9.59 ***

^§^ Number of colonies investigated; * *p* < 0.05; ** *p* < 0.01; *** *p* < 0.001; Other ^a^ = Cattle, buffalo, ducks, small wild mammals; Other ^b^ = Countries other than Thailand and Vietnam; NC = Not calculated due to insufficient data.

**Table 2:** Summary of prevalence estimates (N = 25) of resistance against antimicrobials tested among *Campylobacter* spp. isolates from a total of 18 publications in SEA.

Variable		Total No. Estimates	AMP		CN		C		TE		NAL		CIP		ERY	
			n*	median	n*	median	n*	median	n*	median	n*	median	n*	median	n*	median
Host species	Pig	5	1	51.2	4	10.1	4	2.0	4	84.2	2	89.6	5	84.5	5	66.0
	Poultry	19	10	37.1	14	11.0	9	2.2	13	71.0	11	83.3	17	66.6	17	23.7
Country	Thailand	15	5	31.2	9	0.0	8	0.0	12	77.2	7	79.6	15	81.2	13	50.5
	Vietnam	6	4	57.9	5	16.2	4	9.9	2	73.0	6	92.0	6	44.5	6	62.5
	Others**	4	2	55.7	4	18.8	2	11.2	3	80.9	1	58.3	2	48.1	4	21.9
Total		25	11	40.0	18	11.0	14	2.0	17	76.6	14	83.6	23	70.4	23	46.2

AMP = ampicillin; CN = gentamicin; C = chloramphenicol; TE = tetracycline; NAL = nalidixic acid; CIP = ciprofloxacin; ERY = erythromycin. * n = number of estimates where the antimicrobial was investigated; ** Cambodia, Malaysia, and the Philippines.

**Table 3:** Summary of phenotypic prevalence of AMR in bacteria isolated from aquaculture products (data extracted from eight publications).

Ref.	Country	Species	Host Species	Location	No. Isolates	Prevalence of AMR
[20]	Thailand	*Aeromonas* spp.	Fish	Chicken-fish farm	27	OTC (37%), SMX (19%), ERY (11%), C (7%), CIP (4%)
[20]	Thailand	*Aeromonas* spp.	Fish	Fish farm (no chicken manure)	45	S (22%), OTC (13%), ERY (2%), C (2%), CIP (0%)
[20]	Thailand	*Enterococcus* spp.	Fish	Fish farm using chicken manure	97	S (72%), OTC (75%), ERY (91%), C (8%), CIP (15%)
[20]	Thailand	*Enterococcus* spp.	Fish	Fish farm (no chicken manure)	69	S (31%), ERY (23%), OTC (16%), CIP (6%), C (0%)
[76]	Vietnam	*Edwardsiella ictaluri*	Fish	Diseased fish	64	S (83%), OTC (81%), TMP (73.4%), FLU (8%), OA (6%), ENR (5%), C (0%), NIT (0%), AMX (0%), AMC (0%), KA (0%), CN(0%), NEO (0%), FFC (0%)
[77]	Malaysia and Indonesia	*Streptococcus dysgalactiae*	Fish	Diseased fish	4	OTC (100%), AMP (0%), ERY (0%), FFL (0%), LCM (0%)
[78]	Thailand	*Klebsiella* spp.	Shrimp	Market	67	AMP (100%), TE (100%), BAC (100%), CLI (100%), S (47%), SXT (47%), C (47%), RIF (47%), NAL (12%)
[79]	Vietnam	*Edwardsiella ictaluri*	Fish	Diseased fish	19	SXT (89%), FFC (47%), C (47%), OTC (31%), NIT (0%)
[80]	Vietnam	*Pseudomonas* spp.	Fish	Fish farm	116	AMP (99%), SXT (93%), NIT (90%), NAL (93%), C (89%), TE (30%), S (28%), DOX (25%), CN (16%), KA (12%), NOR (9%), CIP (9%), NEO (3%)
[80]	Vietnam	*Aeromonas* spp.	Fish	Fish farm	92	AMP (94%), SXT (61%), NAL (52%), TE (34%), C (31%), S (31%), NIT (25%), DOX (15%), KA (12%), CIP (8%), CN (6%), NEO (5%), NOR (4%)
[81]	Thailand	*Streptococcus agalactiae*	Fish	Diseased fish	4	AMP (100%), CN (100%), C (100%), ENR (100%), OXA (100%), NIT (100%), PEN (75%), FFC (75%), SXT (50%), ERY (50%)
[82]	Thailand	*Streptococcus agalactiae*	Fish	Diseased fish	144	OA (100%), CN (100%), SMX (100%), TMP (93%), OTC (12%), NOR (2%), LCM (1%), AMP (0%), C (0%), ERY (0%)

Key: AMP = ampicillin, AMX = amoxicillin, AMC = augmentin, BAC = bacitracin, C = chloramphenicol, CIP = ciprofloxacin, CLI = clindamycin, CN = gentamicin, DOX = doxycycline, ENR = enrofloxacin, ERY = erythromycin, FLU = flumequin, FFC = flofenicol, FOM = fosfomycin, KA = kanamycin, LCM = lincomycin, NAL = nalidixic acid, NEO = neomycin, NOR = norfloxacin, NIT = nitrofurantoin, OA = oxolinic acid, OXA = oxacillin, OTC = oxytetracycline, PEN = penicillin, S = streptomycin, SMX = sulfamethoxazole, SXT = co-trimoxazole, TE = tetracycline, TMP = trimethoprim, RIF = rifampicin.

**Table 4:** Antimicrobial resistance (AMR) genes identified in bacterial isolates reported in 33 publications.

Antimicrobial Class	*E. coli*		NTS		Others	
	n	Gene(s) Detected	n	Gene(s) Detected	n	Gene(s) Detected and Host Bacteria
Tetracycline	4	*tet*A, *tet*B, *tet*C	5	*tet*A, *tet*B, *tet*C, *tet*G	11	*tet*A (AC, AP), *tet*B (KL, AC, AP), *tet*D (KL), *tet*L (EN, SS), *tet*M (AP, SD, EN, SS, MRSA), *tet*O (CA, SS), *tet*S (EN), *tet*39 (AC)
Quilonone	2	*gyr*A, *par*C	3	*gyr*A, *gyr*B, *par*C, *par*E,	4	*gyr*A (CA, KL), *gyr*B (CA, KL), *par*C (KL)
	4	*acc*(6)-*Ib*, *qnr*A, *qnr*S, *Oqx*A	1	*qnr*S		
Diaminopyrimidine	7	*dfr*A1, *dfr*A5, *dfr*A10, *dfr*A12, *dfr*A17, *dhfr*17, *dhfr*I, *dhfr*V, *dhfr*XII	8	*dfr*A1, *dfr*A7, *dfr*A10, *dfr*A12, *dfr*A17, *dhfr*XI	1	*dfr*A1, *dfr*A12, *dfr*A21 (PS, AE)
*β*-lactam	5	*bla*_SHV_, *bla*_TEM_, *bla*_CTX-M_	10	*bla*_TEM_, *bla*_PSE-1_, *bla*_OXA-1_, *bla*_OXA-30_	2	*bla*_ROB-1_ (AP), blaZ (MRSA)
	1	MOXM, DHAM	1	*tem*A, *tem*B		
Aminoglycoside	8	*aad*A1, *aad*A2,*aad*A3, *aad*A4, *aad*A5, *aad*A22, *aad*A23, *aad*B	10	*aad*A1, *aad*A2, *aad*A4, *aad*A5, *aad*A22, *aad*B	2	*aad*A1 (PS, AE), *add*A2 (PS, AE), *aad*A9 (CA), aadD (MRSA)
	2	*str*A, *str*B	3	*str*A, *str*B		
	3	*aac*(3)-IV, *aac*A4, *aph*A1, *aph*-(3′)-IA	3	*aac*(3)-IV, *aph*A1-1AB, *aph*A2	2	*aac*-6′-*aph*2″ (EN), acc-aphD (MRSA)
Sulphonamide	5	*sul*1, *sul*2, *sul*3	4	*sul*1, *sul*2, *sul*3	2	*sul*1 (CA, AC), *sul*2 (AC)
Phenicol	4	*cat*A, *cat*B, *cml*A	3	*cat*A, *cat*B, *cml*A, *flor*R	3	*catpI*P501 (EN), *cat*B8 (PS, AE), cat, fexA (MRSA)
Polymixin	3	*mcr-*1				
Lincosamide	2	*lin*F, *lnu*F				
Macrolide					7	23S rRNA (CA), *erm*A (AP, EN), *erm*B (AP, EN, SS, MRSA), *erm*D (BA)
Polypeptide					2	*van*A, *van*C1, *van*C2/3 (EN)

n = Number of publications; AC = *Acinetobacter* spp., AE = *Aeromonas* spp., AP = *Avibacterium paragallinarum*, BA = *Bacillus* spp., CA = *Campylobacter* spp., EN = *Enterococcus* spp., KL = *Klebsiella* spp., PS = *Pseudomonas* spp., MRSA = Methicillin-resistant *Staphylococcus aureus*, SD = *Streptococcus dysgalactiae*, SS = *Streptococcus suis*.

## Supplementary Materials

The following are available online at www.mdpi.com/2079-6382/5/4/37/s1, Table S1: Publications reporting on AMU in terrestrial and aquatic animal farming in SEA, Table S2: The median (and IQR) prevalence of AMR against all tested antimicrobials by country and species among NTS isolates, Table S3: The median (and IQR) prevalence of AMR against all tested antimicrobials by country and species among *E. coli* isolates, Table S4a: Number of isolates of 10 *Salmonella* serovars showing antimicrobial resistance, Table S4b: Prevalence of antimicrobial resistance of Salmonella serovars, Table S5: Phenotypic AMR in organisms other than *E. coli*, NTS and *Campylobacter* spp. in terrestrial animals.
