# Supplementary material for: Antimicrobial Usage and Antimicrobial Resistance in Animal Production in Southeast Asia: A Review
Source: Antibiotics (Basel). 2016 Nov 2;5(4):37. doi: 10.3390/antibiotics5040037 (PMC5187518; doi:10.3390/antibiotics5040037)
Supplement: Supplementary file 1 [file antibiotics-05-00037-s001.zip › Supplementary Materials/Table S1 and S5.pdf]

Antimicrobial Usage and Antimicrobial Resistance in Animal Production in Southeast Asia: A Review

Nguyen T. Nhung, Nguyen V. Cuong, Guy Thwaites and Juan Carrique-Mas

Table S1. Publications reporting on AMU in terrestrial and aquatic animal farming in SEA.

Ref.	Production Species	Year of Study	No. Farms	Country and Region	No. of Classes (and Antimicrobials Used) (Period of usage)	Type of Data	Classes (and Antimicrobials) Used
[21]	Chickens	NR	8	Central region, Thailand, central area	15 (8) (2 months]	Qualitative	**Aminoglycosides** (CN, NEO), **penicillins** (AMP, AMX), **quinolones** (ENR, NOR), **macrolides** (ERY, TY, ROX)**,** **phenicols** (C), **tetracyclines** (TE, CTC, DOX), **sulphonamides** (SPD), **diaminopyrimidine** (TMP)
[20]	Chickens	2009/10	210	Red River Delta, Vietnam	12 (28) (Period not specified)	Qualitative	**Aminoglycosides** (STM, NEO, CN, KA), **penicillins** (PEN, AMP, AMX), **1st generation cephalosporins** (CFL), **quinolones** (ENR, NOR), **macrolides** (ERY, JSM, SRM, TY), **lincosamides** (LCM), **phenicols** (C, THI), **sulphonamides** (SMX, SUD, SQ, SCP, SUG), **tetracyclines** (DOX, OTC, CTC), **pleuromutilins** (TIA), **diaminopyrimidines** (TMP)**,** **polymixins** (CT)
	Pigs				13 (31) (Period not specified)	Qualitative	**Aminoglycosides** (STM, NEO, CN, KA), **penicillins** (PEN, AMP, AMX), **1st generation cephalosporins** (CFD), **3rd generation cephalosporins** (CTX), **quinolones** (ENR, NOR, DAN, FLU), **macrolides** (ERY, KSM, SRM, TY), **pleuromutilins** (TIA), **phenicols** (FFC, C, THI), **sulphonamides** (SMX, SCP, SUG), **tetracyclines** (TE, OTC, CTC, DOX), **lincosamides** (LCM), **diaminopyrimidines** (TMP)**,** **polymixins** (CT)
[16,23]	Chickens	2012/13	208	Mekong Delta, Vietnam	11 (28) (~3 months]	Qualitative/ quantitative	**Aminoglycosides** (STM, NEO, CN, APR, S), **penicillins** (AMP, AMX), **quinolones** (FLU, OA, NOR, ENR), **macrolides** (TY, TIL, ERY, SRM), **phenicols** (FFC, THI), **sulphonamides** (SMX, SUD, SDT, SDM), **diaminopyrimidine** (TMP**)**, **tetracyclines** (TE, CTC, DOX), **polymixins** (CT), **lincosamides** (LCM), **pleuromutilins** (TIA)

Table S1. *Cont.*

Ref.	Production Species	Year of Study	No. Farms	Country and Region	No. of Classes (and Antimicrobials Used) (Period of usage)	Type of Data	Classes (and Antimicrobials) Used
[18]	Chickens		6	Mekong Delta, Vietnam	8 (10) (4 months)	Qualitative/ quantitative	**Aminoglycosides** (CN), **penicillins** (AMX), **1st generation cephalosporins** (CFL), **macrolides** (TY, TIL), **phenicols** (FFC), **quinolones** (ENR, NOR), **polymixins** (CT), **tetracyclines** (DOX)
	Pigs		4		8 (14) (6 months]	Qualitative/ quantitative	**Aminoglycosides** (NEO, CN, KA), **penicillins** (AMX, AMP), **3rd generation cephalosporins** (CEF), **macrolides** (TY, SRM), **phenicols** (C, FFC), **quinolones** (ENR, NOR), **polymixins** (CT), **tetracyclines** (DOX)
[22]	Catfish	2011/12	32	Mekong Delta, Vietnam	11 (17) (Period not specified)	Qualitative	**Aminoglycosides** (APR, KA), **penicillins** (AMP, AMX), **1st generation cephalosporins** (CFL), **quinolones** (CIP, ENR), **polymixin** (CT), **phenicols** (FFC), **diaminopyrimidine** (OMT, TMP), **tetracyclines** (OTC, DOX), **rifamycins** (RIF), **sulphonamides** (SDT, SMX)
	Shrimp	2011/12	34	Several locations, (Thailand)	1	Qualitative	**Tetracyclines** (OTC)
	Tilapia	2011/12	31		2 (2)	Qualitative	**Penicillins** (AMX), **tetracyclines** (OTC)
	Shrimp	2011	34		2 (2)	Qualitative	**Penicillins** (AMX), **quinolones** (NOR)
[17]	Fish and shrimp (several species)	2011	94	Red River Delta and Mekong Delta, (Vietnam)	11 (24)	Qualitative	**Penicillins** (PEN, AMP, AMX), **1st generation cephalosporins** (CFL), a**minoglycosides** (NEO, KA), **diaminopyrimidine (**TMP), **macrolides** (ERY), **phenicols** (FFC, THI), **tetracyclines** (TE, OTC, CTC, DOX), **polymixins** (CT), **quinolones** (ENR, NOR, OF, CIP, FLU), **sulphonamides** (SMX, SUD, SPD), **rifamycins** (RIF)

NR = Not reported; AMP = ampicillin, AMX = amoxicillin, APR = apramyxin, C = chloramphenicol, CEF = ceftiofur, CFD = cefradine, CFL = cefalexin, CIP = ciprofloxacin, CN = gentamicin, CT = colistin, CTC = chlortetracycline, CTX = cefotaxime, DAN = danofloxacin, DOX = doxycycline, ENR = enrofloxacin, ERY = erythromycin, FFC = florfenicol, FLU = flumequin, JSM = josamycin, KA = kanamycin, KSM = kitasamycin, LCM = lincomycin, NEO = neomycin, NOR = norfloxacin, OA = oxolinic acid, OF = ofloxacin, OMT = ormethoprim, OTC = oxytetracycline, PEN = penicillin, RIF = rifampicin, ROX = roxithromycin, S = streptomycin, SCP = sulphachlorpyrazin, SDT = sulphadimethoxine, SDM = sulphadimerazine, SQ = sulphaquinoxaline, SPD = sulphadiazine, SRM = spiramycin, STM = spectinomycin, SUD = sulphadimidine, SUG = sulphaguanidine, SMX = sulfamethoxazole, TE = tetracycline, TIA = tialmulin, TIL = tilmicosin, THI = thiamphenicol, TMP = trimethoprim, TY = tylosin.

Table S5. Phenotypic AMR in organisms other than *E. coli*, NTS and *Campylobacter* spp. in terrestrial animals.

Ref.	Country	Organism	Host	Source	Method of Testing	No. Isolates	Prevalence of AMR
[57]	Indonesia	*Enterococcus* spp.	Chicken	Farm	Agar dilution	116	AMP (0%), C (4.3%), CN (3.5%), ENR (44.0%), ERY (64.7%), KA (49.2%), LCM (74.2%), OTC (73.3%), S (35.4%), VAN (0%)
[57]	Thailand	*Enterococcus* spp.	Chicken	Farm	Agar dilution	70	AMP (0%), C (11.4%), CN (1.4%), ENR (48.6%), ERY (71.4%), %), KA (52.8%), LCM (74.3%), OTC (75.8%), S (28.6%), VAN (0%)
[57]	Vietnam	*Enterococcus* spp.	Chicken	Abattoir	Agar dilution	111	AMP (15.3%), C (25.2%), CN (29.8%), ENR (76.65%), ERY (90.9%), KA (55.9%), LCM (89.2%), OTC (98.2%), S (68.5%), VAN (0%)
[40]	Thailand	*Enterococcus* spp.	Pig	Farm	Disk diffusion	29	C (2.0%), CIP (2%), CLI (90.0%), ERY (80.0%), KA (48.0%), PEN (20.0%), TE (80.0%), S (82.0%), SMX (72.0%)
[84]	Malaysia	VRE	Chicken	Farm		140	No other antimicrobials investigated
[85]	Malaysia	VRE	Chicken	Farm			No other antimicrobials investigated
[87]	Malaysia	VRE	Chicken	Market	Disk diffusion	33	AMP (27.0%), BAC (82.0%), C (61.0%), CAZ (100%), CLT (100%), CN (97.0%), ERY (100%), KA (100%), NAL (100%), NOR (97.0%), PEN (85.0%), S (100%), TE (91.0%)
[86]	Malaysia	VRE	Beef	Market	Disk diffusion	22	AMP (41.0%), BAC (100%), C (69.0%), CAZ (100%), CN (100%), ERY (100%), KA (100%), NAL (100%), PEN (41%), S (100%), SXT (95.4%), TE (100%)
[88]	Thailand	VRE	Pig	Farm	Disk diffusion	52	AMP (53.8%), C (34.6%), CIP (32.7%), CN (15.4%), ERY (61.5%), FOM (3.8%), NIT (1.95%), LZ (11.5%), QDP (13.5%), S (11.5%), RIF (9.6%), TE (86.5%),
[90]	Thailand	MRSA	Pig	Farm	Disk diffusion	5	CLI (100%), ERY (60.0%), FCX (100%), FOM (0%), PEN (100%), SXT (20.0%), VAN (0%)
[89]	Thailand	MRSA	Pig	Farm	Agar dilution and Etest	3	C (100%), CIP(100%), CLI (100%), CN (100%), ERY (66.7%), KA (100%), LZ (0%), STM (0%), SXT (0%), RIF (0%), TE (100%), VAN (0%)

Table S5. *Cont.*

Ref.	Country	Organism	Host	Source	Method of Testing	No. Isolates	Prevalence of AMR
[93]	Thailand	MRSA	Pig	Farm	Disk diffusion	6	AMC (50.0%), C (50.0%), CLI (100%), CN (50.0%), CRO (33.3%), FOX (76.9%), CZ (33.3%), DOX (33.3%), OTC (100%), PEN (100%), SXT (83.3%), TE (100%)
[91]	Thailand	MRSA	Pig	Farm and market	Broth microdilution	11	C (45.5%), CIP (100%), CN (100%), ERY (36.4%), FFC (45.5%), FOX (100%), PEN (100%), S (18.2%), SMX (27.3%), STM (18.2%), SXT (27.3%), TE (100%), TIA (100%), TMP (100%)
[92]	Thailand	MRSA	Farmer and Pig	Farm	Disk diffusion Agar dilution	4	CN (75.0%), CZ (75.0%), ERY (75.0%), FOX (100%), OF (100%), OXA (100%), TE (100%), VAN (0%)
[92]	Thailand	MRSA	Farmer and Pig	Farm	Disk diffusion Agar dilution	40	CN (17.5%), CZ (5.0%), ERY (42.5%), FOX (47.5%), OF (15.9%), OXA (100%), TE (62.5%), VAN (0%)
[96]	Thailand	*Mycoplama gallisepticum*	Chicken	Farm	Broth microdilution	20	ENR (100%), ERY (100%), LIN (0%), OTC (0%), TIA (0%), TY (0%)
[97]	Indonesia	*Haemophilus paragallinarum*	Chicken	Farm	Disk diffusion	14	AMP (7.1%), DOX (35.7%), ERY (78.6%), NEO (71.4%), OTC (57.1%), S (78.6%), SXT (78.6%)
[95]	Thailand	*Avibacterium paragallinarum*	Chicken	Farm	Disk diffusion	18	AMC (0%), AMP (33.3%), AMX (27.8%), CEF (27.8%), CN (5.6%), DOX (38.9%), ENR (27.8%), ERY (77.8%), LCM (100%), NEO (100%), OTC (55.6%), PEN (27.8%), S (11.1%), SXT (66.7%), TY (0%)
[100]	Thailand	*Clostridium perfringens*	Pig	Farm	Broth microdilution	122	AMP (0.8%), BAC (3.2%), CEF (31.1%), CTC (3.2%), DOX (4.1%), ENR (40.2%), ERY (54.9%), LCM (56.6%), OTC (4.1%), TY (13.1%)
[98]	Thailand and Malaysia	*Burkholderia*	Human, animal and soil	Hospital, animals, environment	Disk diffusion	7	AMP (85.7%), BAC (100%), C (0%), CN (100%), CLT (100%), CRO (85.7%), ERY (100%), KA (42.9%), NAL (0%), S (100%), TE (0%)

Table S5. *Cont.*

Ref.	Country	Organism	Host	Source	Method of Testing	No. Isolates	Prevalence of AMR
[99]	Malaysia	*Burkholderia*	Human, animals	Laboratory	Disk diffusion	6	AMP (100%), KA (100%), CN (100%)
[94]	Vietnam	*Streptococcus suis*	Pig	Farm	Disk diffusion and E-test	45	C (26.7%), CIP (0%), ERY (51%), PEN (0%), VAN (0%), TE (100%)

Note: MRSA = methicillin resistant *Staphylococcus aureus*, MRS = methicillin resistant *Staphylococci*, VRE = vancomycin resistant *Enterococci*, NA = not available; Key: AMP = ampicillin, AMC = augmentin, AMX = amoxicillin, BAC = bacitracin, C = chloramphenicol, CLI = clindamycin, CLT = cephalothin, CIP = ciprofloxacin, CTC = chlortetracycline, CN = gentamicin, CZ = cefalozin, CTX = cefotaxime, CRO = ceftriaxone, CEF = ceftiofur, DOX = doxycycline, ENR = enrofloxacin, ERY = erythromycin, FOM = fosfomycin, FOX = cefoxitin, FCX = flucloxacillin, KA = kanamycin, NAL = nalidixic acid, NEO = neomycin, NIT = nitrofurantoin, NOR = norfloxacin, LCM = lincomycin, LZ = linezolid, OTC = oxytetracycline, OF = ofloxacin, OXA = oxacillin, PEN = penicillin, QDP = quinupristinedalfopristin, RIF = rifampin , SMX = sulphamethoxazole, S = streptomycin, STM = spectinomycin, SXT = co-trimoxazole, TIA = tiamulin, TE = tetracycline, TMP = trimethoprim, TY = tylosin, VAN = vancomycin.

© 2016 by the authors; licensee MDPI, Basel, Switzerland. This article is an open access article distributed under the terms and conditions of the Creative Commons by Attribution (CC-BY) license (http://creativecommons.org/licenses/by/4.0/).
